# Engineering of Bioenergy Crops: Dominant Genetic Approaches to Improve Polysaccharide Properties and Composition in Biomass

**DOI:** 10.3389/fpls.2020.00282

**Published:** 2020-03-11

**Authors:** Andrew G. Brandon, Henrik V. Scheller

**Affiliations:** ^1^Department of Plant and Microbial Biology, University of California, Berkeley, Berkeley, CA, United States; ^2^Feedstocks Division, Joint BioEnergy Institute, Emeryville, CA, United States; ^3^Environmental Genomics and Systems Biology Division, Lawrence Berkeley National Laboratory, Berkeley, CA, United States

**Keywords:** lignocellulosic biomass, dedicated bioenergy crops, genetic engineering, cellulose, hemicellulose, cell walls, carbohydrate active enzymes, polysaccharides

## Abstract

Large-scale, sustainable production of lignocellulosic bioenergy from biomass will depend on a variety of dedicated bioenergy crops. Despite their great genetic diversity, prospective bioenergy crops share many similarities in the polysaccharide composition of their cell walls, and the changes needed to optimize them for conversion are largely universal. Therefore, biomass modification strategies that do not depend on genetic background or require mutant varieties are extremely valuable. Due to their preferential fermentation and conversion by microorganisms downstream, the ideal bioenergy crop should contain a high proportion of C6-sugars in polysaccharides like cellulose, callose, galactan, and mixed-linkage glucans. In addition, the biomass should be reduced in inhibitors of fermentation like pentoses and acetate. Finally, the overall complexity of the plant cell wall should be modified to reduce its recalcitrance to enzymatic deconstruction in ways that do no compromise plant health or come at a yield penalty. This review will focus on progress in the use of a variety of genetically dominant strategies to reach these ideals. Due to the breadth and volume of research in the field of lignin bioengineering, this review will instead focus on approaches to improve polysaccharide component plant biomass. Carbohydrate content can be dramatically increased by transgenic overexpression of enzymes involved in cell wall polysaccharide biosynthesis. Additionally, the recalcitrance of the cell wall can be reduced via the overexpression of native or non-native carbohydrate active enzymes like glycosyl hydrolases or carbohydrate esterases. Some research in this area has focused on engineering plants that accumulate cell wall-degrading enzymes that are sequestered to organelles or only active at very high temperatures. The rationale being that, in order to avoid potential negative effects of cell wall modification during plant growth, the enzymes could be activated post-harvest, and post-maturation of the cell wall. A potentially significant limitation of this approach is that at harvest, the cell wall is heavily lignified, making the substrates for these enzymes inaccessible and their activity ineffective. Therefore, this review will only include research employing enzymes that are at least partially active under the ambient conditions of plant growth and cell wall development.

## Introduction

Lignocellulosic plant biomass represents the largest renewable source of organic carbon on earth. Organic carbon that can be converted into a wide variety of compounds, including high-energy liquid fuel, thereby curbing our dependence on non-renewable sources and limiting the net production of carbon dioxide. The bulk of plant biomass is contained in the cell wall, specifically the thick secondary cell walls (SCW) of the vasculature and fiber tissues. The cell wall has evolved a highly complex and rigid structure to resist the mechanical forces of growth and protect the plant from various stresses. The most valuable component of the cell wall, from a bioenergy perspective, is the 6-carbon sugar glucose comprising the linear polysaccharide cellulose. Produced and extruded into the developing cell wall by cellulose synthase (CesA) complexes at the plasma membrane, individual cellulose chains coalesce through hydrogen bonding to form crystalline cellulose microfibrils. In all vascular plants, the SCW is composed of cellulose microfibrils embedded in a matrix of the aromatic polymer lignin. Hemicellulose like xylan coat the cellulose microfibrils and can form covalent linkages with cell wall proteins, lignin, and other hemicelluloses (Gírio et al., 2010; Scheller and Ulvskov, 2010; Meents et al., 2018). This natural complexity makes the deconstruction and recovery of usable sugars costly and resource intensive.

The prevailing sources of biofuels to date have been sucrose from crops like sugarcane or sugar beet and starch from corn. While much simpler and cheaper to process, their sustainability at a larger scale is dubious considering all are also major food and forage crops. The “food vs. fuel” competition for arable land could drive up the price of food and have negative socioeconomic impacts. Therefore, modern approaches to sustainable bioenergy emphasize the development of dedicated bioenergy crops that can be grown on marginal land (Himmel and Bayer, 2009; Cai et al., 2011). Ideally, these crops will be fast-growing perennials, producing the maximum biomass per unit land over multi-year cycles and minimizing nutrient input needs (Sanderson and Adler, 2008). The additional constraints of varying soil quality, water availability, and average temperature mean biomass productivity will vary depending on where the crops are grown and that no single engineered species can meet bioenergy demands around the world (Somerville et al., 2010; Chen and Peng, 2013). For example, in tropical and sub-tropical climates, elephant or Napier grass (*Pennisetum purpureum*) produces more biomass per hectare annually than any other vegetation. Grasses like *Miscanthus* × *giganteus*, sorghum, and switchgrass (*Panicum virgatum*), and tree species like poplar, aspen, and willow are capable of producing large amounts of biomass in temperate regions like Europe and the United States (Heaton et al., 2008; Guidi et al., 2013; Junior et al., 2016). While the massive bioethanol productivity of Brazil comes from sugarcane sucrose, the lignocellulosic biomass left over could additionally be engineered for better bioenergy conversion. Crassulacean acid metabolism plants like *Agave* spp. have the highest water use efficiency of all plants, making them attractive bioenergy feedstock crops for cultivation on the increasing percentage of the world’s land area considered arid (<800 mm of rainfall per year) (United Nations Environment Programme, 2007; Borland et al., 2009).

The wide variety of potential feedstock species means efforts to improve their quality through biotechnology should be as broadly useful as possible. Research into the fundamental cell biology of plants and the organisms that degrade them has revealed the causes of biomass recalcitrance and a variety of approaches to reduce it. Significant improvements in biomass have been demonstrated by suppressing or eliminating the expression of various genes related to the biosynthesis of specific polysaccharides (Biswal et al., 2015, 2018; Loqué et al., 2015; Kalluri et al., 2016; Wang et al., 2016; Willis et al., 2016; Bhatia et al., 2017; Donev et al., 2018; Li et al., 2019b). However, the usefulness of the same approaches in a range of bioenergy crops can be limited by their comparative genetic complexity. Therefore, this review will focus on cell wall polysaccharide engineering strategies that act independent of genetic or genomic context, primarily via the overexpression of recombinant or native enzymes. Importantly, we include only enzymes that are active *in planta* concurrent with cell wall development. The engineering of plants to accumulate hyperthermophilic or inactive cell wall-degrading enzymes has been well reviewed in other works (Mir et al., 2014; Damm et al., 2016; Park et al., 2016).

## Modulation of Polysaccharide Biosynthesis

### Cellulose

Two major features of an “ideal” bioenergy crop are high cellulose content and a high ratio of C6:C5 sugar residues comprising the polysaccharide content. Increasing cellulose biosynthesis is an important goal for engineering efforts because cellulose consists entirely of the C6 sugar glucose and the genes involved in its biosynthesis are relatively well-understood and conserved among land plants. Secondary cell wall (SCW) cellulose, which accounts for the bulk of cellulosic biomass in bioenergy-relevant crops, is synthesized at the plasma membrane by three, non-redundant cellulose synthase (CesA) proteins CesA4, CesA7, and CesA8 (McFarlane et al., 2014). Overexpression of CesAs is, therefore, a logical approach to generating transgenic plants enriched in cellulose. However, attempts to overexpress SCW CesAs in aspen and barley resulted in co-suppression and decreased cellulose content (Joshi et al., 2011; Tan et al., 2015). Overexpression of SCW CesA4 and CesA6 driven by a maize ubiquitin promoter in switchgrass also resulted in decreased cellulose, even though co-suppression did not occur (Mazarei et al., 2018). See Table 1 for a summary of studies discussed in this review. The reason for the decrease in cellulose in the study by Mazarei and coworkers is unclear, but the plants exhibited reduced growth. The study would suggest that careful consideration of promoters may be critical for the success of overexpressing SCW CesAs in crops. Greater success has been demonstrated in Arabidopsis (*Arabidopsis thaliana*) by overexpression of either of the primary cell wall (PCW) CesAs, *CesA2*, *CesA5* and *CesA6* (Hu et al., 2018). CesA2, CesA5, and CesA6 are each functional in a PCW cellulose synthase complex including CesA1 and CesA3. Transgenic plants overexpressing one of the three genes had 29–37% increase in crystalline cellulose content. Expression of both *CesA1* and *CesA3* was significantly higher in transgenic lines, indicating the possible secretion and activity of PCW CesA complexes even during SCW development. Transgenic lines also had more xylan and a slight, but significant, increase in lignin, potentially counteracting the benefits of the increase in cellulose. However, enzymatic saccharification efficiency of the transgenic biomass was not reported and further work is required to determine the usefulness of PCW *CesA*-overexpression. A clever approach to increase SCW cellulose content that avoids the negative effects of co-suppression has been employed to generate transgenic sugarcane (*Saccharum* spp.). While plants, many bacteria and some fungi can produce cellulose, only one group of animals is known to do so: the marine invertebrates of subphylum *Tunicata*, otherwise known as sea squirts (Matthysse et al., 2004; Kimura and Itoh, 2007). A cellulose synthase cDNA from *Ciona savignyi* (*CsCesA*) was used to create transgenic sugarcane overexpressing a functional form of the protein (Ndimande, 2013). Use of this divergent gene sequence did not cause any co-suppression and the internode cellulose content was increased by up to 31%. Additionally, all tissues of *CsCesA*-overexpressing sugarcane lines had increased saccharification efficiency, with increases of 39% and 28% in young and mature internodes, respectively. Since sucrose is the primary source of bioenergy potential in sugarcane, total soluble sugars were also measured. Transgenic lines yielded up to 25% more, contrary to intuition that sucrose content would be depleted by increased conversion to UDP-glucose (UDP-Glc) for cellulose production. It could be that this depletion acts as a signal to source organs to increase production and/or transport of sucrose to sink organs. It has previously been demonstrated that induced depletion of sucrose from sugarcane stems increases photosynthetic productivity and phloem loading from leaves (McCormick et al., 2009; Wang et al., 2013). In the non-cellulosic polysaccharide fraction of the cell wall, Ndimande (2013) also observed increased a 56% increase in glucose. 22% increase in galactose and a 53% increase in galacturonic acid. The author attributes the increase in glucose to increased biosynthesis of mixed-linkage glucan, which uses UDP-Glc as its substrate. An increased mixed-linkage glucan deposition driven by higher UDP-Glc has previously been reported in barley starch mutants (Christensen and Scheller, 2012). While sugarcane accumulates large amounts of soluble sucrose, the majority of the carbohydrate mass of the plant is still the cell wall, making it an interesting model for the study of carbon flux to cell wall biosynthesis.

**TABLE 1:** Summary of dominant approaches to modify biomass polysaccharide composition.

Engineered species	Transgene expressed	Effects on biomass composition and conversion	References
*A. thaliana*	*AtCesA2*, *AtCesA5*, *AtCesA6*	(+) 29–37% Cel	Hu et al., 2018
	*GhCOBL9A*	(+) 59% Cel	Niu et al., 2018
	*AtGalS1::AtUGE2::AtURGT1*	(+) 80% Gal	Gondolf et al., 2014; Aznar et al., 2018
	*pSAG12:OsCSLF6*	(+) non-Cellulosic Glc	Vega-Sánchez et al., 2015
	*AtIRX10*^G283D^, *AtIRX10*^E293Q^	(−) 39–55% Xyl	Brandon et al., 2019
	*AtSBD123*	(+) 76% FW; (+) 50% HC; (+) 30% Pec; (+) 100% non-crystalline Cel; (+) 28% IVD	Grisolia et al., 2017
	*pEST:PcPL1*	(+) 90–100% SE, post-induction	Tomassetti et al., 2015
	*pSAG12:AnPGA2*	(+) 50–100% SE, post-senescence	Tomassetti et al., 2015
	*AtPMEI-2*	(+) 50% SE; (+) 68% DW	Lionetti et al., 2010
*Populus* spp.	*GhSuSy*	(+) 2–6% Cel; (+) CrI	Coleman et al., 2009
	*PdDUF266A*	(+) 17–34% DW; (+) 37% Cel; (+) 13% Cel DP; (+) 38% SE	Yang et al., 2017a
	*AnAXE1*	(+) 26% SE	Pawar et al., 2016, 2017
	*PdDUF231A*	(+) 8–21% Cel; (−) 6–8% lignin	Yang et al., 2017b
	*AaXEG2*	(+) DW; (+) 81% SE	Kaida et al., 2009; Park et al., 2004
	*PtPL1*	(+) SE	Biswal et al., 2014
Tobacco	*PsnSuSy1/2*	(+) 18% Cel; (−) 28% lignin	Wei et al., 2015; Li et al., 2019a
	*AcCel5*	(+) 10–15% SE	Brunecky et al., 2011
	*TrCel5*	(−) FW; (−) Cel	Klose et al., 2015
	*ClEXPA1/2*	(+) FW; (+) 30−50% Cel	Wang et al., 2011
	*AnPGA2*	(+) 100% SE; (−) 50−84% FW	Lionetti et al., 2010
*P. virgatum*	*PvCesA4*, *PvCesA6*	(−) DW; (−) 6−33% Cel; (+) 2−12% Xyl	Mazarei et al., 2018
	*OsAT10*	(+) 40% Cel	Li et al., 2018
*O. sativa*	*OsSuSy3*	(+) 15−26% Cel; (+) 11−13% HC	Fan et al., 2017, 2019
	*OsGH9B1*, *OsGH9B3*	(+) 63% SE	Huang et al., 2019
	*OsARAF1*, *OsARAF3*	(−) 19–25% Ara; (−) 28−34% Cel	Sumiyoshi et al., 2013
	*OsAT10*	(+) 8−19% Cel; (+) 40% SE	Bartley et al., 2013
*Saccharum* spp.	*CsCESA*	(+) 31% Cel; (+) 28–39% SE; (+) 25% Suc; (+) 56% non-Cel Glc; (+) 22% Gal; (+) 53% GalA	Ndimande, 2013
*F. arundinacea*	*AnFAE*	(+) 10–14% IVD	de Buanafina et al., 2008, 2010; Morris et al., 2017

*Strong, constitutive promoters were used unless otherwise indicated. Cel, cellulose; Ara, arabinose; Glc, glucose; Gal, galactose; GalA, galacturonic acid; Suc, sucrose; Xyl, xylose; HC, hemicellulose; Pec, pectin; FW, fresh weight; DW, dry weight; DP, degree of polymerization; CrI, cellulose crystallinity index; SE, saccharification efficiency; IVD, *in vitro* digestibility.*

**FIGURE 1:**
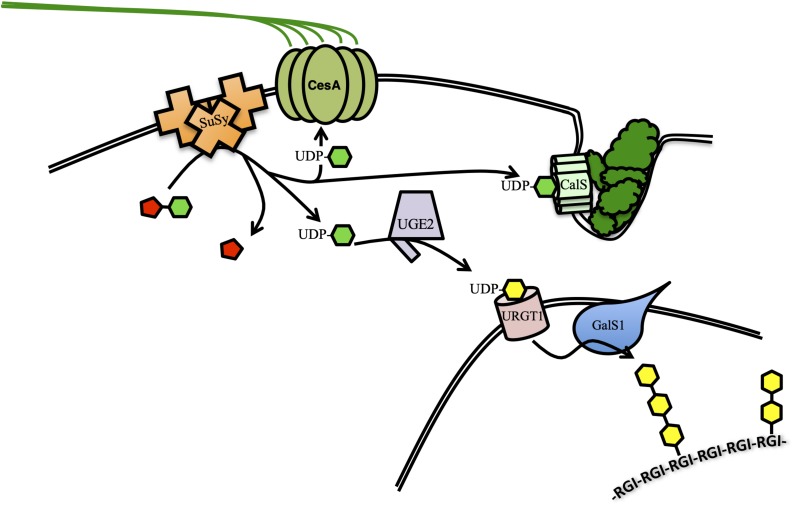
Schematic representation of the role of sucrose synthase (SuSy) in increasing the biosynthesis of several C6-sugar polysaccharides. SuSy isoforms can be localized to the cytoplasm, or tightly associated with the plasma membrane, some interacting directly with CesA. All catalyze the reversible conversion of sucrose into fructose (red) and UDP-Glc (green). UDP-Glc is the substrate for cellulose synthesis by CesA complex or callose synthesis by CalS at the plasma membrane. UDP-Glc can also be converted to UDP-Gal (yellow) by UGE1, imported to the Golgi by URGT1, and used to synthesize the galactan side chains of pectic RGI.

Increased cellulose biosynthesis has been accomplished by overexpressing the enzyme responsible for producing its substrate. Sucrose synthase (SuSy) proteins catalyze the cleavage of sucrose to fructose and UDP-Glc, which is the sole substrate for the biosynthesis of glucans like cellulose, callose and mixed-linkage glucans. Evidence suggests that some SuSy isoforms interact directly with the CesA complex, channeling UDP-Glc directly to cellulose biosynthesis (Figure 1; Fujii et al., 2010; Stein and Granot, 2019). Overexpression of cotton (*Gossypium hirsutum*) *SuSy* in hybrid poplar (*Populus alba* × *grandidentata*) resulted in small (2–6%) increases in cellulose and increased cellulose crystallinity (CrI) compared to controls (Coleman et al., 2009). CrI reflects the degree of hydrogen bonding between individual cellulose chains and is a primary contributor to biomass recalcitrance by reducing the proportion of cellulose exposed to cellulolytic enzymes (Hall et al., 2010). Overexpression of the hybrid poplar (*Populus simonii* × *Populus nigra)* gene *PsnSuSy2* in tobacco led to 25% thicker cell walls, containing up to 18% more cellulose and decrease in lignin of up to 28%, when compared to controls. Additionally, the degree of cellulose crystallinity (CrI) was reduced by 9–11% (Wei et al., 2015; Li et al., 2019a). Overexpression of the endogenous *PvSUS1* in switchgrass resulted in up to 14% more biomass, but in contrast to the studies of tobacco and poplar, lignin was increased rather than decreased (Poovaiah et al., 2015). Cellulose crystallinity was not reported, but saccharification was reduced, perhaps due to the increased lignin. A similar approach in transgenic rice (*Oryza sativa*) lines overexpressing *OsSuSy3* under a SCW-specific or a constitutive promoter resulted in dramatic improvements in multiple bioenergy-relevant characteristics (Fan et al., 2017, 2019). The effects on cell wall composition were similar using either promoter. Total plant biomass was only slightly increased in transgenic lines, but microscopic analysis of the cell wall revealed a 68% increase in cell wall thickness compared to controls. The increase in cell wall thickness corresponded to a 15–26% increase in cellulose and 11–13% increase in hemicelluloses, while lignin content was not significantly changed. Cellulose from transgenic plants had a 7–10% reduction in CrI, but an increased cellulose degree of polymerization (DP) by 8–15%. DP, like CrI, is negatively correlated with saccharification efficiency. After pretreatment, transgenic lines yielded 13–23% total sugars, leading to a 20–49% greater ethanol yield and 22% higher conversion efficiency when compared to controls. The 2017 and 2019 studies distinguish themselves by investigating secondary effects of *OsSuSy3* overexpression on two important agronomic traits, respectively: lodging resistance and susceptibility to pathogen or insect attack. Lodging is a complex trait and crop susceptibility to lodging can dramatically reduce yield and increase the cost of harvesting. Lodging Index is a combination of several measurements taken from the plant to determine its physicomechanical strength and resistance to lodging. All four independent transgenic lines overexpressing *OsSuSy3* showed increases of 17–50% in Lodging Index compared to controls, suggesting they could grow robustly and be high-yielding in the field. In the more recent study by Fan and coworkers, *OsSuSy3* transgenic lines were observed to be less susceptible to a number of biotic stresses including bacterial blight, fungal rice blast, and herbivory by the brown planthopper. These resistances correlated with a significant increase in callose deposition upon initiation of infection or pest attack. Since both callose and cellulose are synthesized from UDP-Glc, it is possible that *OsSuSy3* overexpression could also stimulate callose biosynthesis (Figure 1). The β-(1,3)-Glc bonds in callose make it much less crystalline than cellulose, thus more amenable to saccharification. It would be intriguing to see if this accumulation of callose phenotype could be exploited, perhaps by triggering an immune response at senescence in order to rapidly accumulate low-recalcitrance callose before harvest. These works demonstrate that overexpression of a single gene involved in carbohydrate flux can simultaneously improve a variety of traits important in bioenergy crops.

In addition to the *CesA* genes, several other genes that are important for, or even critical to, cellulose biosynthesis have been identified based on the phenotypes of their loss-of-function mutants. Their gene products often lack structural similarity to CesA proteins or even to glycosyltransferases in general, so the roles they play in cellulose biosynthesis can be difficult to determine. Despite this, a few studies have demonstrated that the overexpression of such proteins can lead to dramatic improvements to biomass composition and recalcitrance. A specific example is the engineered overexpression of a gene, *Domain of Unknown Function-266A (DUF266A)*, in poplar (*Populus deltoides)* and Arabidopsis (Yang et al., 2017a). DUF266-containing proteins are only present in land plants and are categorized as “non-classifiable GT” (Lao et al., 2014), although they are distantly related to GT family 14. GT14s have been characterized as having a range of activities, from arabinogalactan biosynthesis in plants to protein *O*-glycosylation in animals (Cantarel et al., 2009; Hansen et al., 2012; Knoch et al., 2013). The rice gene *Brittle Culm 10 (OsBC10)* is the only previously characterized gene encoding a DUF266-containing protein. *OsBC10* encodes a Type II transmembrane Golgi protein and loss-of-function mutant plants were dwarfed with reductions in cellulose, and increases in xylose and lignin, indicating a role in cell wall biosynthesis (Zhou et al., 2009). The transgenic poplar lines overexpressing *PdDUF266A* generated by Yang et al. (2017a) had increased total biomass of 17–34% and cellulose content up to 37% greater than wild type. Their cellulose DP was also increased by 13%, although the CrI was not significantly altered. Total sugar release after enzymatic saccharification was increased in *PdDUF266A*-overexpressors by 38% compared to controls. These effects could be indirect, since several genes involved with SCW cellulose biosynthesis were found to be significantly upregulated. How a protein without a predicted function, residing in the Golgi, is able to alter gene expression is not currently understood. However, there is a more direct effect *PdDUF266A* overexpression may have had on cell wall composition (Yang et al., 2017a). When probing transverse section of the *osbc10* mutant, Zhou et al. (2009) found a dramatic decrease in signal using antibodies specific to arabinogalactan proteins. Compositional analysis revealed *osbc10* mutants to have a 72% reduction in cell wall arabinogalactan proteins compared to controls. While it was not measured in the study, it is possible that overexpression of *PdDUF266A* modified the arabinogalactan protein profile of the cell wall, which could cause alterations in the organization or interaction of various wall polysaccharides. PdDUF266A could also be involved directly in the glycosylation of CesA proteins or other proteins critical to cellulose synthesis like KORRIGAN and COBRA. The latter two proteins play roles in microfibril deposition and require posttranslational *N*-glycosylation to be fully functional (Roudier et al., 2005; Liebminger et al., 2013). Recently, a *COBRA-like* gene from cotton (*Gossypium hirsutum*), *GhCOBL9A*, was overexpressed in Arabidopsis, leading to dramatic increases in total biomass and cellulose content (Niu et al., 2018). The cell walls of cotton fibers consist almost entirely of cellulose and are an interesting model for high-level cellulose production. Of the 33 identified *COBL* genes in the cotton genome, *COBL9A* was expressed highly during SCW development and co-expressed with SCW *CesA* genes. COBL proteins are secreted and have a glycosylphosphatidylinositol anchor to the plasma membrane. COBLs also have a carbohydrate-binding domain (CBM) that preferentially binds to crystalline cellulose. COBLs are required for cellulose production, believed to direct the orderly deposition of nascent cellulose microfibrils but are not components of the cellulose synthase complex (Liu et al., 2013). Niu et al. (2018) found that overexpression of *GhCOBL9A* in Arabidopsis produced plants that grew taller and contained up to 59% more cellulose. The expression of three SCW *CesA* genes, *CesA4*, *CesA7* and *CesA8*, were also dramatically increased, approximately 10-fold higher than controls. Interestingly, examination of transverse stem sections of transgenic plants revealed increased cellulose and wall thickening, not only in fiber and vessel cells, but also in pith and parenchyma cells. These tissues normally contain only cells with a thin primary wall. Increasing cellulose production in these cell types or even engineering them to deposit a SCW would be an interesting strategy with potential to increase total cellulose content in bioenergy crops.

### Hemicelluloses and Pectin

Although it is by far the most abundant, cellulose is not the only C6-sugar polysaccharide in the cell wall. Co-overexpression of *Galactan Synthase1* (*GalS1*) with the gene encoding an enzyme that provides its substrate, *UDP-Glc/UDP-Gal-4-Epimerase2* (*UGE2*), in Arabidopsis increased galactose content in the cell wall by up to 80% (Gondolf et al., 2014). GalS1 transfers galactose to β-1,4-galactan side chains of the rhamnogalacturonan I (RGI) backbone domain of pectin. Overexpression of *UDP-Rha/UDP-Gal Transporter1 (URGT1*), responsible for transport of UDP-Gal from the cytoplasm to the Golgi, in combination with *GalS1* and *UGE2* further boosted galactose content in the stems to four times the levels in wild-type plants (Figure 1; Aznar et al., 2018). In these plants the transgenes were driven by SCW-promoters and β-1,4-galactan accumulated in secondary walls where it is not normally found, except for specialized walls in gelatinous fibers. Another hexose polysaccharide found in the cell walls of grasses is mixed-linkage glucan. The accumulation of significant amounts of mixed-linkage glucans has been successfully engineered in Arabidopsis through overexpression of *Cellulose synthase-like F6* from rice (*OsCslF6*) (Vega-Sánchez et al., 2015). The cell walls of *OsCslF6*-overexpressor lines contained four times more non-cellulosic glucose. Additionally, the saccharification efficiency of plants producing mixed-linkage glucans was increased by 42% compared to wild type. The variety of linkages between glucose residues in mixed-linkage glucans make it much more amorphous and soluble than cellulose, and thus more amenable to saccharification. The greater solubility of mixed-linkage glucan means it is easily extracted from the biomass post-harvest, which in turn exposes more of the cellulose surface to hydrolytic enzymes. While high-level production of mixed-linkage glucans is, therefore, an attractive trait to engineer into bioenergy crops, the choice of promoter appears to be critical. Overexpression of *CslF6* in barley, Arabidopsis and tobacco with constitutive or SCW-specific promoters had severely adverse effects on plant growth (Burton et al., 2011; Vega-Sánchez et al., 2015). The successful outcomes of the study by Vega-Sanchez and coworkers depended on the use of the promoter of *A. thaliana SENESCENCE ASSOCIATED GENE-12* (*pAtSAG12*), a promoter active only during senescence.

In conjunction with increasing C6 sugars like glucose and galactose, a reduction in C5 sugars like xylose is also desirable in the cell walls of dedicated bioenergy crops. Mutants in xylan biosynthesis have been identified in model species, but genetic redundancy and greater genomic complexity in bioenergy crops make full knockouts or knockdowns difficult to generate (Brown et al., 2007, 2009; Peña et al., 2007; Wu et al., 2010; Lee et al., 2012; Chen et al., 2013; Mortimer et al., 2015). In all cases, significant decreases in xylan biosynthesis in mutants was accompanied by severely reduce growth. Recent work from our lab has successfully demonstrated a novel approach to reducing the amount of a specific polysaccharide with a protein-level, dominant knock-down of xylan biosynthesis (Brandon et al., 2019). The gene *Irregular Xylem 10 (IRX10)* and its partially redundant homolog *Irregular Xylem 10-like (IRX10-L)* encode GT47 enzymes that unambiguously exhibit xylan β-(1,4)-xylosyltransferase activity in recombinant systems (Jensen et al., 2014; Urbanowicz et al., 2014). However, other proteins, IRX9 and IRX14 in particular, play critical, likely structural roles in the functional xylan synthase complex (Wu et al., 2010; Ren et al., 2014). As of this publication, no crystal structure of a GT47 protein has been resolved, so the catalytic site of IRX10 is unknown. However, potentially important amino acid residues can be inferred to be involved in catalysis based on their very high degree of conservation in evolutionarily divergent species. Two of these residues, Gly-283 and Glu-293 drastically reduced or eliminated enzymatic activity when mutated. By overexpressing the mutated *IRX10* (*dnIRX10*) genes in wild-type Arabidopsis, the mutant isoforms (IRX10^G283D^ and IRX10^E293Q^) out-competed the native IRX10 for its place in the proposed xylan synthase complex. The stems of *dnIRX10*-overexpressing plants had reductions in xylose content of up to 55% compared to wild type. While xylan has detrimental effects on recalcitrance and biomass conversion, it is critical to the strength of vessel and fiber SCWs, and mutants in xylan biosynthesis exhibit severe growth defects due primarily to collapsed xylem vessels and impaired water and nutrient transport (Brown et al., 2005, 2009; Lee et al., 2007; Wu et al., 2010). The phenotypes of *dnIRX10*-overexpressing lines, unsurprisingly, mimicked those of xylan knockout mutants. Previous work in our lab has demonstrated that the growth phenotype of xylan biosynthetic mutants can be rescued by expression of a functional copy of the gene under the control of a vessel cell-specific promoter (Petersen et al., 2012). Therefore, much of the xylose reduction could be maintained without growth penalty if expression of the *dnIRX10* transgene was abrogated in vessel cells or if a strong, fiber-specific promoter were used.

## Polysaccharide Modification

### Cellulose

**FIGURE 2:**
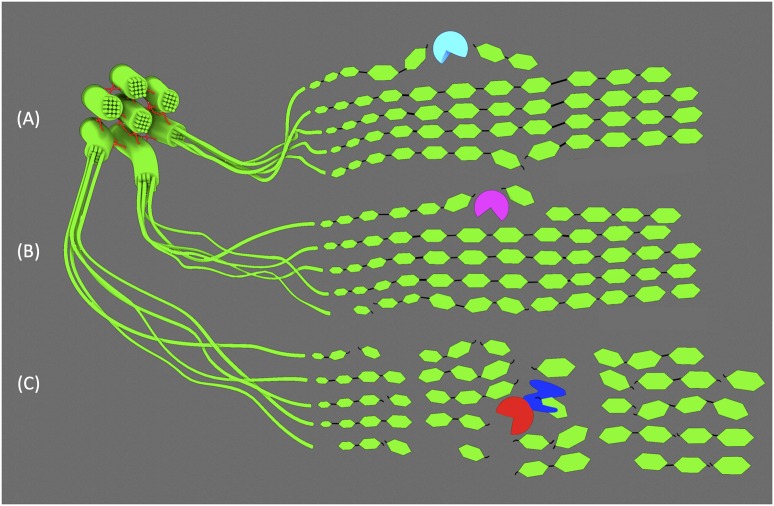
GH cuts to cellulose microfibrils. **(A)** GH9B1 and GH9B3 and **(B)** thermophilic, CBM-truncated AcCel5A make relatively few cuts to superficial strands in the microfibril. **(C)** Mesophilic TrCel5A binds to cellulose via its CBM (blue), while the endoglucanase domain (red) makes cuts with significantly higher frequency due to its temperature optimum being similar to plant growth conditions.

Biomass quality can be improved by transgenic expression of enzymes that modify the polysaccharides of the cell wall prior to its full maturation. Plants express various glycosyl hydrolases (GH) for building and remodeling the wall in many different tissues and stages of development (Barnes and Anderson, 2018). The GH9 β-1,4-endoglucanases in plants are hypothesized to play a role in cellulose remodeling and biosynthesis by cutting specifically between glucose residues of a cellulose chain. The GH9 subfamily B is distinguished by the absence of either a transmembrane domain or carbohydrate binding module (CBM) (Hayashi et al., 2005). Recent work in rice has demonstrated that overexpression of two native GH9Bs (*OsGH9B1* and *OsGH9B3*) dramatically improved biomass quality without significant growth or developmental defects (Huang et al., 2019). The cell wall composition was unchanged compared to the control and the plants grew normally. However, the transgenic lines exhibited a 18–23% decrease in cellulose DP and an 11–23% reduction in CrI. After pretreatment and enzymatic saccharification of the biomass, both *GH9B1* and *GH9B3*-overexpressors released 63% more reducing sugars than control lines. Since total cellulose content in transgenic lines was unchanged, the authors posit that the enzymes directly decreased DP and CrI by cleaving microfibrils, thereby increasing accessibility to cellulases by increasing the number of exposed cellulose ends (Figure 2A).

Genes encoding GH enzymes from lignocellulose-degrading fungi can also be used to engineer bioenergy crops for heterologous expression. Work using the thermophilic endoglucanase gene from *Acidothermus cellulolyticus AcCel5A* suggests that even minimal cuts in cellulose chains can have dramatic effects on microfibril crystallinity, increasing enzymatic digestibility and sugar yield (Brunecky et al., 2011; Donohoe et al., 2017). AcCel5A is known to have high activity at high temperatures on synthetic substrates *in vitro*, but to be ineffective at degrading mature cell walls. Theorizing that *in planta* expression might be more effective, transgenic lines of maize and tobacco producing apoplast-targeted AcCel5A were generated. The high temperature optimum of the enzyme was a deliberate and important consideration, as it suggested activity would be low enough to avoid deleterious effects on normal plant growth. Indeed, both tobacco and maize plants grew normally and were less recalcitrant to bioconversion, yielding 10–15% more glucose from cellulose than untransformed plants under the same conditions (Brunecky et al., 2011). Importantly, adding recombinant AcCel5A to post-harvest cell wall material could not replicate these results, indicating that enzyme activity concurrent with cellulose production and deposition is key. In order to better understand the role of AcCel5A and build on prior work in maize and tobacco, the same endoglucanase gene was transformed into Arabidopsis to engineer overexpressor lines (Figure 2B; Donohoe et al., 2017). As in previous experiments using maize and tobacco, the composition of the cell wall was unchanged in transgenic plants and they grew normally. However, after closer inspection of the cell wall with scanning electron microscopy, large voids, pockets, and other structural irregularities were observed in cell walls of *AcCel5A*-overexpressors. Interestingly, these features mimicked the characteristics of electron micrographs of plant biomass after various chemical pretreatments. Thus, *in planta* expression of *AcCel5A* is able to mimic the role of pretreatment to increase the cellulose surface area accessible to hydrolytic enzymes. This presents the obvious benefit of likely reducing the chemical and enzyme input necessary downstream.

The choice of endoglucanase and the organism it is derived from is important. Overexpression and apoplast-targeting in tobacco of a similar GH from the mesophilic fungus *Trichoderma reesei*, TrCel5A, caused severe growth defects and a significant decrease in cellulose content (Klose et al., 2015). Presumably, TrCel5A would be more active than AcCel5A since *T. reesei* evolved in similar conditions to plants. Additionally, TrCel5A possesses a CBM that was truncated from the AcCel5A used by Brunecky et al. (2011). CBMs are known to facilitate hydrolytic activity and reduce CrI by physically disrupting the hydrogen bonds between cellulose chains (Figure 2C; Abramson et al., 2010). This disruptive effect could be exploited for the improvement of bioenergy crops, since CrI is strongly correlated with biomass recalcitrance. However, relatively few studies have been published exploring the engineered overexpression of CBMs, or CBM-containing proteins like expansins, to this end. Expansins are cell wall proteins that disrupt the intermolecular hydrogen bonds of cellulose and hemicelluloses, promoting the flexibility and extensibility of the primary cell wall. They play critical roles in primary growth by maintaining the delicate balance between turgor pressure and cell wall integrity that drive cell expansion (Cosgrove, 2000, 2005). However, some expansins are specifically expressed in vessel and fiber cells during SCW development. Two such expansin genes from Chinese fir (*Cunninghamia lanceolata)* have been cloned and used to engineer overexpression lines in tobacco (Wang et al., 2011). Both *Expansin-A1 and Expansin-A2 (ClEXPA1 and ClEXPA2)* overexpressing lines grew taller and had thicker stems than wild type. The cell walls of xylem cells were 1.13 to 1.45 times thicker in transgenic plants and when the composition of stem cell wall material was analyzed, they contained 30–50% more cellulose. It is also interesting to explore the effects that CBMs alone can have on cell wall architecture. In Arabidopsis, the coding sequence of the CBM of *Starch Synthase III (SSIII)* was overexpressed and targeted to the cell wall (Grisolia et al., 2017). Though starch and cellulose are very different in structure, previous work indicated that the concatenated triplicate of CBMs (collectively referred to as SBD123) from SSIII had preferential affinity for the linear portions of the starch molecule. Thus, the rationale behind the work was that it may also bind to linear cellulose and modify the crystallinity of microfibrils. Like the tobacco plants expressing *ClEXPA* genes, Arabidopsis plants overexpressing *SBD123* grew significantly larger than untransformed lines. The average cell area was increased by 40% and dry biomass weight by 76%. Unlike the *ClEXPA*-expressing plants, Grisolia et al. (2017) observed a 27% reduction in cell wall thickness and similar amounts of cellulose in transgenic Arabidopsis stems. This suggests SBD123 loosened the components of the cell wall, stretching the cell wall to cover a greater cell volume. However, hemicellulose and pectin contents were significantly higher (50% and 30%, respectively) and dilute acid hydrolysis of cell wall material from transgenic plants yielded almost twice as much glucose. Dilute acid is generally insufficient to hydrolyze crystalline cellulose and there is relatively little xyloglucan in Arabidopsis stems. Thus, it is reasonable to conclude that the glucose released is derived from crystalline cellulose made amorphous by the disruptive effects of SBD123-cellulose interaction. Finally, an *in vitro* rumen digestibility assay determined biomass from transgenic plants was 28% easily digested than that of control plants. However, other attempts to engineer plants expressing CBMs have been less successful with variable results and sometimes negative effects on growth and cell wall recalcitrance (Safra-Dassa et al., 2006; Obembe et al., 2007; Keadtidumrongkul et al., 2017).

### Xylan

Xylan is the most abundant hemicellulose in most bioenergy crops and contributes significantly to biomass recalcitrance by enveloping cellulose microfibrils and forming covalent linkages to lignin and other hemicelluloses in the cell wall. Thus, modifications to xylan are important engineering goals in the development of dedicated bioenergy crops. The xylan backbone is β-(1,4)-linked xylose residues, many of which can be mono- or di-acetylated, and is decorated to varying degrees with glucuronic acid (GlcA), 4-*O*-methyl glucuronic acid (MeGlcA) and, in grasses, arabinose (Ara) side chains (Rennie and Scheller, 2014). Arabinose side chains can be further modified by esterification with ferulic acid or *p*-coumaric acid moieties. The amount and distribution of these decorations to the xylan backbone determine the properties of the polysaccharide *in muro*. Several engineering strategies targeting these side chains have been successfully employed to reduce biomass recalcitrance.

**FIGURE 3:**
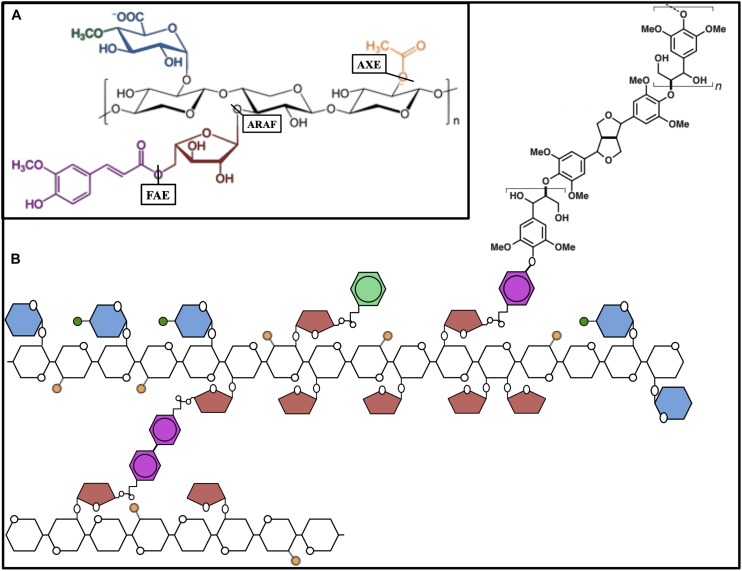
The hemicellulose xylan and interactions. **(A)** Schematic molecular structure of xylan module with β-(1,4)-linked xylose residues (black) of the xylan backbone that are substituted with acetyl (orange), arabinose (red), and glucuronic acid (blue) residues. Arabinose is partially esterified with ferulic acid (magenta) and glucuronic acid is often 4-*O*-methylated (green). Acetyl xylan esterase (AXE), arabinofuranosidase (ARAF), and ferulic acid esterase (FAE) indicating the bonds they hydrolyze. **(B)** Schematic representation of the xylan chain, xylan-xylan diferulate cross-linking, and ferulic acid-mediated lignin polymerization.

Approximately 40–60% of xylose residues comprising xylan are acetylated at the O-2 or O-3 position (Busse-Wicher et al., 2014), and reductions in acetylation correlate positively with conversion efficiency. Additionally, acetic acid released from xylan during pretreatment is a strong inhibitor of microbial fermentation downstream. To engineer a reduction in xylan acetylation, an acetyl xylan esterase from the lignocellulose-degrading fungus *Aspergillus niger* (*AnAXE1*) was overexpressed in Arabidopsis (Figure 3) (Pawar et al., 2016). After observing beneficial effects, the same gene was used to generate transgenic overexpressors in hybrid aspen (*Populus tremula L.* × *tremuloides Michx*) (Pawar et al., 2017). The transgenic trees developed normally with approximately 10% less xylan acetylation. This reduction in acetylation had the secondary effect of reducing the average xylan DP. Since no changes in xylan biosynthesis were observed, the authors propose that xylan with reduced acetylation may be more susceptible to cleavage by endogenous cell wall hydrolases. These modifications to xylan led to a 26% increase in saccharification efficiency compared to controls without pretreatment. The positive effect of *AnAXE1*-overexpression was confirmed after acid pretreatment of the biomass. Acid pretreatment removed xylan, and the difference in saccharification efficiency between transgenic and control lines was greatly reduced. The effects of xylan acetylation on recalcitrance, however, are not yet fully understood. The overexpression of a gene encoding a DUF231-containing protein in poplar (*PdDUF231A*) had beneficial effects on biomass composition, despite increasing xylan acetylation by 8% (Yang et al., 2017b). It is likely that the effect on xylan acetylation is an indirect one, though, since the PdDUF231A is phylogenetically more closely related to PMR5, a pectin acetyltransferase (Chiniquy et al., 2019), than to the known xylan acetyltransferases. *PdDUF231A*-overexpressing lines exhibited an 8–21% increase in total cellulose content and 6–8% reduction in lignin, while cell wall content of other cell wall sugars was unchanged. Metabolite analysis of the transgenic lines additionally suggested the increase in carbon flux to cellulose biosynthesis came at the expense of lignin biosynthesis. While the precise role of *PdDUF231A* is unknown, the combination of beneficial biomass changes it produced makes it an interesting subject of further research. Despite the successful studies on reducing xylan acetylation in plants, it is clear that xylan acetylation is important for plant growth and development (Xiong et al., 2013). Therefore, we should expect that the successful engineering of reduced xylan acetylation will depend on careful choice of promoters and target cell types.

The xylans of grasses are extensively arabinosylated, allowing the formation of many cross-linkages with other components in the cell wall and directly affecting the characteristics of cellulose microfibrils (Li et al., 2015). Reducing the number of xylan arabinose side chains has great potential to improve the biomass quality of grasses, perhaps the most important group of dedicated biomass crops. Two native arabinofuranosidase genes from rice (*OsARAF1* and *OsARAF3*), were used to engineer rice lines accumulating high levels of the protein in the cell wall (Figure 3; Sumiyoshi et al., 2013). Transgenic lines had 19–25% less arabinose in matrix cell-wall polysaccharides and a 28–34% increase in cellulose content. It is likely both factors combined led to a 46–70% increase in enzymatic sugar release compared to wild type. Many xylan arabinose side chains are esterified with ferulic acid, which can form extensive diferulate covalent bonds with other glucuronoarabinoxylans. Ferulic acid can also act as a nucleation site for the polymerization of lignin (Grabber et al., 2004; Terrett and Dupree, 2019). These properties make ferulic acid a significant contributor to the recalcitrance of grass biomass. Diferulate cross-linking and the free-radical nucleation of lignin happen spontaneously and non-enzymatically. Thus, enzymes severing the connection between ferulic acid and arabinose are particularly useful for biomass engineering. Fortunately, we can again draw from ingenuity of lignocellulose-degrading fungi. *In planta* expression of a variety of ferulic acid esterases (FAEs) has demonstrated beneficial effects to reduce recalcitrance and increase biomass digestibility, but in many cases there are trade-offs to be considered. Expression of *Aspergillus nidulans* FAE in Arabidopsis and *Brachypodium distachyon* enhanced saccharification but increased susceptibility to fungal pathogens (Pogorelko et al., 2011; Reem et al., 2016). In alfalfa (*Medicago sativa*), expression of *A. niger* FAE did reduce arabinoxylan feruloylation, but unexpectedly led to increased lignification and decreased digestibility (Badhan et al., 2014). The authors noted, however, that saccharification efficiency did increase compared to wild type after chemical delignification, possibly due to a reduction of inter-arabinoxylan cross-linking. The beneficial effect of *in planta* expression of FAEs has been particularly well studied in tall fescue (*Festuca arundinacea)*, an important animal forage crop (de Buanafina et al., 2008, 2010). As fescue is typically studied for its nutritional value to livestock, an *in vitro* model using rumen microorganisms was used to assess cell wall digestibility and *AnFAE*-overexpressors demonstrated a 10–14% increase in digestibility. More recent work with fescue suspension culture cells showed that significantly less time was required for rumen microbes to digest the cell wall from cells overexpressing *AnFAE* than for control cells (Morris et al., 2017). Despite the use of very different analytical techniques, it is reasonable to believe these changes to cell wall composition could translate in bioenergy crops to improvements in enzymatic saccharification. Importantly, this increase in digestion rate could not be replicated by exogenous application of recombinant AnFAE to control cell wall material. Much like the previously described transgenics expressing endoglucanases, the beneficial effects are most significant when the enzyme is active during cell wall development. The observed negative side effects of FAE expression such as increased susceptibility to pathogens could perhaps be mitigated by restricting expression of the transgene in the tissues most affected by the stress, like epidermal cells. Other labs have found success in rice and switchgrass using an alternative approach to reduce ferulate crosslinking in the cell wall by out-competing ferulic acid for arabinosyl side chains on xylan (Bartley et al., 2013; Li et al., 2018). The BAHD-type acyltransferase OsAT10 from rice is a putative *p*-coumaroyl transferase that increases *p*-coumaric acid esterification of arabinose moieties of xylan. Although it is also a phenylpropanoid-derived hydroxycinnamic acid, *p*-coumaric acid does not undergo oxidative coupling to monolignols in the polymerization of lignin (Marcia, 2009). In *OsAT10*-overexpressing lines of rice and switchgrass, the ratio of *p*-coumaroyl to feruloyl esterification of arabinoxylan increased by 150% and 75%, respectively. In rice, *OsAT10* overexpression had the additional effect of increasing cell wall glucose by 8–19% compared to wild type. Transgenic lines of both rice and switchgrass developed normally and both demonstrated an increase of up to 40% in total sugar yield after enzymatic saccharification.

### Other Polysaccharides

While most studies have focused on cellulose and xylan, some studies have targeted other polysaccharides, which are clearly important in the development of SCWs despite their lower abundance. Overexpression of *Aspergillus aculeatus* xyloglucanase gene (*AaXEG2*) in poplar (*Populus alba*) led to increased growth and cellulose deposition and up to 81% more glucose released after enzymatic hydrolysis (Park et al., 2004; Kaida et al., 2009). Pectin modification is likewise a useful strategy as shown in the knock-down studies mentioned above (Biswal et al., 2015, 2018; Li et al., 2019b). The modification of pectin by overexpression of a pectate lyase gene from *Populus trichocarpa* (*PtPL1*) in hybrid aspen (*Populus tremula* × *tremuloides*) resulted in improved saccharification (Biswal et al., 2014). The overall composition of the biomass did not differ significantly from wild-type plants, indicating that the enzyme might act by loosening interactions between matrix wall components and increasing accessibility to cell wall-degrading enzymes. The homogalacturonan domain of the pectin backbone and/or the extent of its methyl-esterification have been targets for *in planta* modification. An *A. niger* polygalacturonase gene (*AnPGA2*) was used to engineer overexpressors in Arabidopsis and tobacco (Lionetti et al., 2010). While transgenic lines had a twofold increase in saccharification efficiency, they were also severely stunted in growth with 50–84% reduction in total biomass. This detrimental effect has been described previously, wherein it was demonstrated that despite the use of a strong promoter, the only plants that survived to be studied accumulated AnPGA2 at very low levels (Capodicasa et al., 2004). A polygalacturonase-inhibiting protein (PGIP) produced by the common bean plant (*Phaseolus vulgaris*) has been identified and well described to eliminate PGA2 activity (Leckie et al., 1999). If the growth defects of PGA2-transgenics were determined to be tissue- or developmental stage-dependent, it would be interesting to include PGIP with a promoter specific to that tissue or developmental stage. Then, the dramatic improvements to saccharification could possibly be engineered into bioenergy crops without sacrificing biomass yield. One study has explored controlled pectin modification by generating transgenic Arabidopsis lines expressing a pectate lyase from *Pectobacterium carotovorum* (*PcPL1*) and the *A. niger* polygalacturonase (*AnPGA2*) (Tomassetti et al., 2015). The β-estradiol inducible promoter (pEST) and the senescence promoter (pSAG12) were used to drive expression of *PcPL1* and *AnPGA2*, respectively. Tissue collected from *pEST:AnPGA2* transgenic plants post-induction yielded almost twice as much glucose as wild-type or non-induced plants. However, some lines expressed *AnPGA2* even in the absence of the inducer and suffered diminished growth. In contrast, all *pSAG12:AnPGA2* transgenic lines grew normally. Expression of the gene was undetectable in plants until they reached 7 weeks old, at the beginning of senescence, and mirrored the expression of the native *SAG12* gene. Stem and leaf biomass from 4 to 7-week-old *pSAG12:PGA2* plants and evaluated for saccharification efficiency. The amount of glucose released by cellulase digestion was comparable between 4-week-old *pSAG12:PGA2* and wild type plants. In contrast, significantly more glucose was released from *pSAG12:PGA2* plants compared to wild type after 7 weeks of growth. The increase was almost twofold from leaf tissue, and about 50% from stems.

Homogalacturonan is extensively methylesterified as it is synthesized and secreted. Once it reaches the cell wall, pectin methylesterases remove many of these methyl groups, revealing carboxylic acid moieties that subsequently form linkages with various components of the cell wall. Free carboxyl groups of de-methylesterified homogalacturonan can additionally form rigid, calcium-mediated “egg box” structures with adjacent stretches of homogalacturonan (Atmodjo et al., 2013). Therefore, maintaining the methylesterification of homogalacturonan could be a viable approach to reducing cell wall recalcitrance. In the previously referenced study by Lionetti et al., pectin methylesterase inhibitor genes from Arabidopsis (*AtPMEI-2*) and kiwi (*Actinidia chinensis*, *AcPMEI*) were overexpressed in Arabidopsis and wheat (*Triticum durum*), respectively (Lionetti et al., 2010). Saccharification efficiency of *AtPMEI-2* overexpressing Arabidopsis stem increased by 50%, while wheat plants overexpressing *AcPMEI* had 2.5-fold greater saccharification efficiency than controls. In contrast to polygalacturonase-overexpressing plants, the total biomass of Arabidopsis plants overexpressing *AtPMEI-2* was greater than wild type by 68%. This increase was not observed in *AcPMEI* overexpressing wheat plants, suggesting differing impacts of homogalacturonan methylesterification between monocots and dicots. The role of minor matrix polysaccharides in the development and structure of SCWs is poorly understood, but the successful results suggest that this would be a fertile ground for further research.

## Conclusion and Future Perspectives

Plant biotechnological research can make a major contribution to meeting perhaps humanities’ most common and critical goals. That is, mitigating the effects of climate change by reducing our net production of carbon dioxide and developing an alternative to the fundamentally unsustainable fossil resources on which we have become dependent. While traditional breeding techniques will be important in the development of dedicated bioenergy crops, genetic engineering allows for significant modifications to biomass to be made much more quickly. As our fundamental knowledge of plant cell biology and the regulation of cell wall development continues to grow, so too will our ability to approach the perfect balance of maximizing yield and quality while minimizing recalcitrance and deleterious side effects. Here we have described a number of dominant genetic engineering strategies to improve plant biomass for bioenergy conversion. Though they may have only been demonstrated in one or two species, they have potential for broad usability in the wide variety of dedicated bioenergy species currently being researched. In several of the studies we have reviewed here, the results observed in different species were somewhat contradictory. For example, overexpression of CesAs or Susy in different species resulted in both increased or decreased cellulose and lignin depending on the particular study. This suggests that direct translation of an engineering strategy from a model plant or from one bioenergy crop to another is not entirely straightforward. The reasons for these contradictory results are not clear. They could be due to differences in the physiology and different cell wall types of the various species. However, we find it more likely that differences in the promoters used in the various studies, including their spatial and temporal expression profiles can explain many of the observed differences. Studies that resolve the results obtained with a larger set of promoters in a single species are required and would help to better predict outcomes in future studies. Future studies also need to determine the impact of environmental factors on the outcomes. If indeed the transgene expression profiles are very important, then we may also expect that the effect on plant development, biomass composition and recalcitrance could be strongly influenced by growth conditions. For the practical deployment of approaches such as those reviewed here, it is very important to determine if observations in the laboratory and greenhouse translate to the field condition, and to understand the mechanisms responsible where differences are observed.

## Author Contributions

AB and HS developed the idea of the work and wrote the manuscript. AB illustrated Figures 1, 3.

## Conflict of Interest

AB and HS are inventors on a patent application related to the work described here on xylan reduction.
